# Iodine nutrition with North Atlantic living: the Faroese adolescents

**DOI:** 10.1017/jns.2022.111

**Published:** 2023-02-08

**Authors:** Herborg Líggjasardóttir Johannesen, Stig Andersen, Stine Linding Andersen, Kári Rubek Nielsen, Pál Weihe, Maria Skaalum Petersen, Anna Sofía Veyhe

**Affiliations:** 1 Department of Endocrinology and Medicine, The National Hospital of the Faroe Islands, Torshavn, Faroe Islands; 2 Center of Health Science, Faculty of Health Science, University of the Faroe Islands, Torshavn, Faroe Islands; 3 Department of Clinical Medicine, Aalborg University, Aalborg, Denmark; 4 Arctic Health Research Centre, Aalborg University Hospital, Aalborg, Denmark; 5 Greenland Centre for Health Research, University of Greenland, Nuuk, Greenland; 6 Department of Clinical Biochemistry, Aalborg University Hospital, Aalborg, Denmark; 7 Department of Gastroenterology and Medicine, The National Hospital of the Faroe Islands, Torshavn, Faroe Islands; 8 Department of Occupational Medicine and Public Health, The Faroese Hospital System, Torshavn, Faroe Islands

**Keywords:** Artic society, Faroe Islands, Health survey, Iodine status, Urinary iodine excretion

## Abstract

Iodine nutrition is critical for human health. While iodine excretion was low within the recommended range among adult Faroese, younger generations tend to abandon local foods. Such changes raise a concern about iodine intake, which led us to perform this first study of iodine nutrition among teenagers in the North Atlantic islands. We used samples from a nationwide collection of urine samples in 14-year-olds following iodine fortification of salt in 2000. Urine was analysed for iodine and creatinine to adjust for dilution by iodine/creatinine, and a food frequency questionnaire was used to record the intake of iodine-rich foods. The 129 participants yielded a 90 % precision of the estimated iodine nutrition level. The median urinary iodine concentration (UIC) was 166 μg/l (bootstrapped 95 % confidence interval 156–184 μg/l). The median creatinine-adjusted UIC was 132 μg/g (bootstrapped 95 % CI 120–138 μg/g). Fish and whale meat dinners were more frequent among residents of villages compared with the capital: median fish dinners, 3 *v.* 2 per week (*P* = 0⋅001), and whale meat, 1 *v.* 0⋅4 per month (*P* < 0⋅001). UIC decreased with fewer fish dinners (*P* = 0⋅03). Our study demonstrated that Faroese teenagers were iodine-replete. The changing dietary habits emphasise the need for continuous monitoring of iodine nutrition and surveying iodine deficiency disorders.

## Graphical abstract

**Figure:**
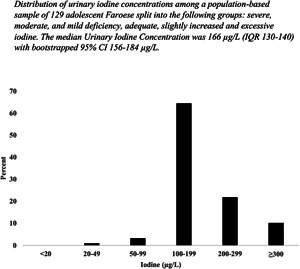

## Introduction

Iodine is an essential micronutrient required for the synthesis of thyroid hormones. It is a determinant of thyroid disease, with low and high iodine intakes raising the disease risk^(^^1–3)^. Severe iodine deficit may cause cretinism^(^^2–4)^, with iodine deficiency being the single most important preventable cause of developmental brain damage worldwide^(^^3,5)^. Conversely, excessive iodine intake can cause hyperthyroidism, hypothyroidism and goitre^(^^2,6)^. In addition, iodine nutrition influences lifespan^(^^7^^)^ and thyroid cancer risk^(^^8)^. Consequently, the World Health Organisation (WHO) recommends monitoring iodine nutrition in all populations^(^^4^^)^, and inadequate dietary intake is a global concern^(^^9)^.

Iodine-rich food items include fish and other seafood, milk and dairy products^(^^10,11)^, and the intake of these food items can support a sufficient iodine nutrition level^(^^12–14)^. People living in coastal areas often rely on a diet dominated by marine food items. An environment unfavourable to terrestrial food production pushes people to rely on marine food items. One example is living on the small islands in the North Atlantic, such as the Faroe Islands.

The first data on iodine nutrition among adult Faroese was from a survey in 2011–12 and reported a median urinary iodine concentration (UIC) of around 101 μg/l, which classified Faroese within but in the lower end of the recommended range for adequate iodine nutrition^(^^15)^. However, younger generations consume less traditional seafood than the previous generations. Thus, the continuous dietary changes raise concerns about iodine nutrition status among younger Faroese and vulnerable groups such as pregnant women.

The present study is the first to assess data on iodine nutrition in Faroese adolescents. We measured iodine in urine from a representative sample of Faroese adolescents around the time of salt iodisation. We aimed to establish baseline data indicating an association with dietary habits from data on relevant food items.

## Experimental methods

### Ethical approval

This study was conducted according to the guidelines laid down in the Declaration of Helsinki, and all procedures involving human subjects were approved by the Scientific Ethical Review Committee (reference number 11.15-2000003/3) and the Danish Data Protection Authority (‘Datatilsynet’ – reference number 2001-41-0814). Participation was voluntary, and informed consent was obtained from all subjects and the participants’ parents.

### Area of investigation

The Faroe Islands are a cluster of eighteen islands constituting an archipelago in the North Atlantic Ocean. The population is approximately 54 000^(16)^. The Faroe Islands are a part of the Nordic countries with similar language, welfare system, ways of life and dietary intake. However, additional traditional Faroese cuisine includes fish and seabirds available due to the proximity to the North Atlantic Ocean. Moreover, traditional Faroese foods include whale meat, blubber, gut tallow, Faroese puffins, local lamb, dried mutton, potatoes and a few fresh vegetables^(17)^. Imported food is mainly Scandinavian cuisine and some grain products from Great Britain^(^^16)^. An important Faroese speciality is pilot whale meat and blubber. ‘Grindadráp’ is a cultural heritage drive-hunting type involving herding pilot whales into shallow bays to be stuck, slain and shared in the community.

Denmark started mandatory iodisation of all salt in the year 2000^(^^18)^. Since the Faroese import most of the edible salt from Denmark, albeit with non-iodised salt also available, an impact on the Faroe Islands is most likely parallel to that of Greenland^(^^14)^. However, there was no regulation or monitoring programme for the Faroe Islands.

### Participants, physical examination and questionnaire

A population-based birth cohort was established on the Faroe Islands consisting of births in 1986–7. The cohort covered 75⋅1 % of all deliveries^(^^19)^. The primary objective of the cohort was to examine prenatal exposure to environmental contaminants. Four follow-up examinations have been performed^(^^19,20)^. The present study includes the 129 participants who donated a spot urine sample at follow-up in 2000/2001 when they were aged 14 years. The participants answered questionnaires, underwent physical examination and donated spot urine samples.

The adolescents’ parents completed a self-administered questionnaire on past medical history, current health status and social factors. In addition, the parent filled in a food frequency questionnaire (FFQ). The FFQ did not include any time frame, and the consumption was based on intake per week or month. The FFQ had the following four dietary questions: ‘How many fish dinners does the child consume weekly’; ‘How many meat meals does the child consume weekly’; ‘How many whale meat dinners does the child consume monthly’; ‘Does the child eat whale blubber (yes/no)’.

The physical examination included measurement of height and weight. Body mass index (BMI) was calculated as weight in kilograms divided by height in metres squared. No participant was treated for thyroid disease, while fourteen were on medication for asthma or allergy. Six commented on health issues: one with ureter constriction, four had previous treatment for asthma or allergy and one was previously treated for epilepsy. None of these conditions influences iodine nutrition.

### Sampling procedures

During the investigation, urine samples were collected and stored in iodine-free containers at −80°C at the Department of Occupational Medicine and Public Health, the Faroe Islands, until analyses were performed in 2003.

### Assay procedures

We determined the iodine concentration in urine by the Sandell–Kolthoff reaction modified after Wilson and van Zyl^(^^21)^. The principle is detecting the catalytic role of iodine in reducing ceric ammonium sulphate in the presence of arsenous acid after pre-treatment with alkaline ashing^(^^22)^. Creatinine in urine was measured using a kinetic Jaffé method^(^^23)^. We calculated the ratio of iodine/creatinine (UI/Cr) to adjust for dilution, as creatinine is excreted in urine at a relatively constant rate^(^^24)^. This adjustment reduces the variations in UIC caused by variations in the urinary volume due to differences in fluid intake^(^^24)^. The iodine laboratory at Aalborg University Hospital (Denmark) is experienced in handling the assay^(^^7,12,14,15,22,25–27)^ successfully participated in the programme to ensure the quality of urinary iodine procedures (the EQUIP programme). We analysed the samples in random order, including internal and external controls.

### Statistical analyses and sample size consideration

Results are presented as median, interquartile range (IQR) and bootstrapped 95 % CI. The non-parametric 95 % confidence intervals (CIs) around the median were obtained using the bootstrap technique (*n* 1000)^(^^28)^.

Continuous variables were analysed for normal distribution by visual inspection of QQ plots and the Kolmogorov–Smirnov test. We tested for differences between groups using the Mann–Whitney *U* test and Pearson's *χ*^2^ test as appropriate. Iodine was positively skewed and therefore log-transformed (log_10_ *x*) before explored relationships using univariate linear regression models for the following variables: sex; fish intake (up to two times per week *v.* 3+ times per week); whale meat (up to once per month *v.* 2+ times per month); whale blubber intake (no *v.* yes); residence (capital *v.* village); BMI; healthy weight (<85th percentile *v.* ≥85th percentile) and smoking (no *v.* yes). Whale blubber intake, BMI, healthy weight and smoking were not included in the multivariate analysis based on *P* > 0⋅5. Variables from the univariable linear regression with a *P*-value <0⋅5 were included in the multivariable regression model (sex, whale meat intake, mother and father's educational level, and fish intake). We saw an interaction between fish intake and place of living, and residence was removed from the model as fish intake was a stronger predictor of the contribution to UIC. The final model meets the requirements for linear regression as the residual distribution showed close to normal, and even distribution was seen in histogram and QQ plots. Also, we found no residual correlation (Durbin–Watson test = 1⋅9) or collinearity (all VIF values <1⋅4). No outliers were removed for the descriptive data.

We chose a 5 % level of significance. The sample size aimed for a 10 % precision range with 95 % confidence in a population^(^^29)^. Data were analysed using the Statistical Program for Social Science (version 25.0; SPSS Inc, Chicago, IL, USA).

## Results

### Study population characteristics

Altogether 129 participants were included in the study. The distribution of sex was equal (*P* = 0⋅9). There was a weekly intake of fish and whale meals among all participants, and two in five reported blubber intake in general (Table 1). There were no sex differences in dietary frequency intake of fish (*P* = 0⋅9), meat (*P* = 0⋅5), whale meat (*P* = 0⋅8) or whale blubber (*P* = 0⋅2). The participants living in the rural areas (*n* 80) consumed significantly more fish- and whale meat dinners than participants living in the capital (*n* 48): the median intake was 3 (IQR 2–5) fish dinners per week *v*. 2 (IQR 1–3), *P*_(MW)_ = 0⋅001, and 1 (IQR 0⋅8–2) whale meat dinners per month *v*. 0⋅4 (IQR 0–1), *P*_(MW)_ < 0⋅001. Conversely, participants living in the capital recorded a median intake of 4 (IQR 3–5) meat dinners (whale meat excluded) per week *v*. 3 (IQR 2–4) for rural residents, *P*_(MW)_ < 0⋅001. Nearly 48 % of capital-living teenagers reported consuming whale blubber regularly compared with 35 % of the rural adolescents, *P*_(Chi)_ = 0⋅2.

**Table 1.** Descriptive characteristics of the study population (*n* 129)

Variables	Groups	Mean (sd*) or *n* (%)
Boys		64 (49⋅6)
Living in a capital area^†^		48 (37⋅2)
Mothers educational level^‡^	Unskilled	75 (58⋅1)
	Short education	31 (24⋅0)
	Bachelor or higher	19 (14)
Body mass index (BMI)^§^		20⋅5 (3⋅1)
	Healthy weight (<85th p)	110 (85⋅3)
	Overweight (≥85th p)	19 (14⋅7)
Smoking (yes and occasionally)^||^		8 (6⋅3)
Fish meals (≥3 dinners per week)		72 (55⋅8)
Whale meat dinners (≥2 dinners per week)^¶^		28 (21⋅7)
Blubber intake (yes)**		51 (39⋅5)

* sd, standard deviation.

† One was missing.

‡ Four were missing.

§ BMI min−max: 15⋅2–30⋅5.

|| Two were missing.

¶ Eleven were missing.

** Three were missing.

### Iodine in urine

**Fig. 1.**
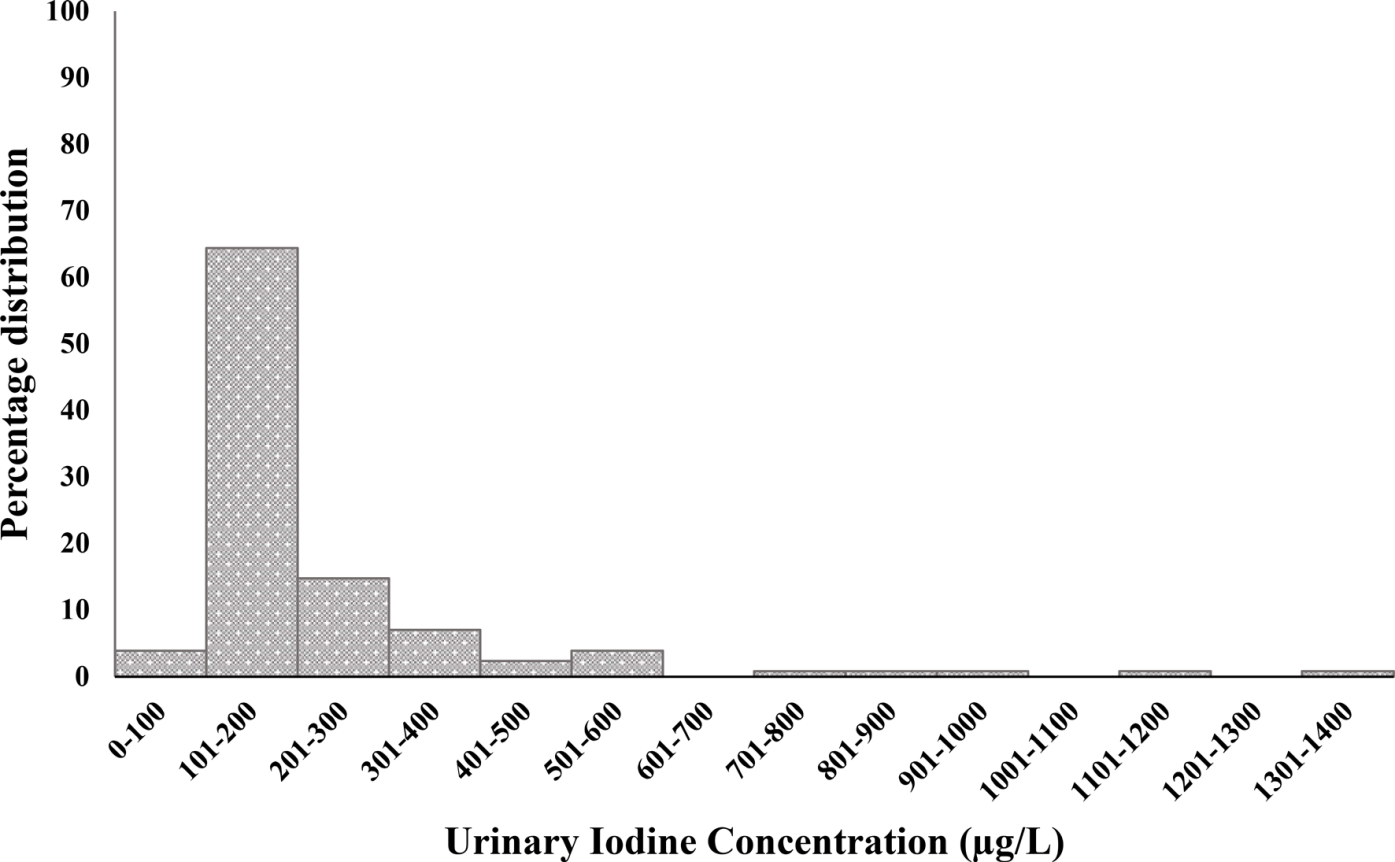
Frequencies of urinary iodine concentrations (μg/l) among a population-based sample of 129 adolescent Faroese.

**Fig. 2.**
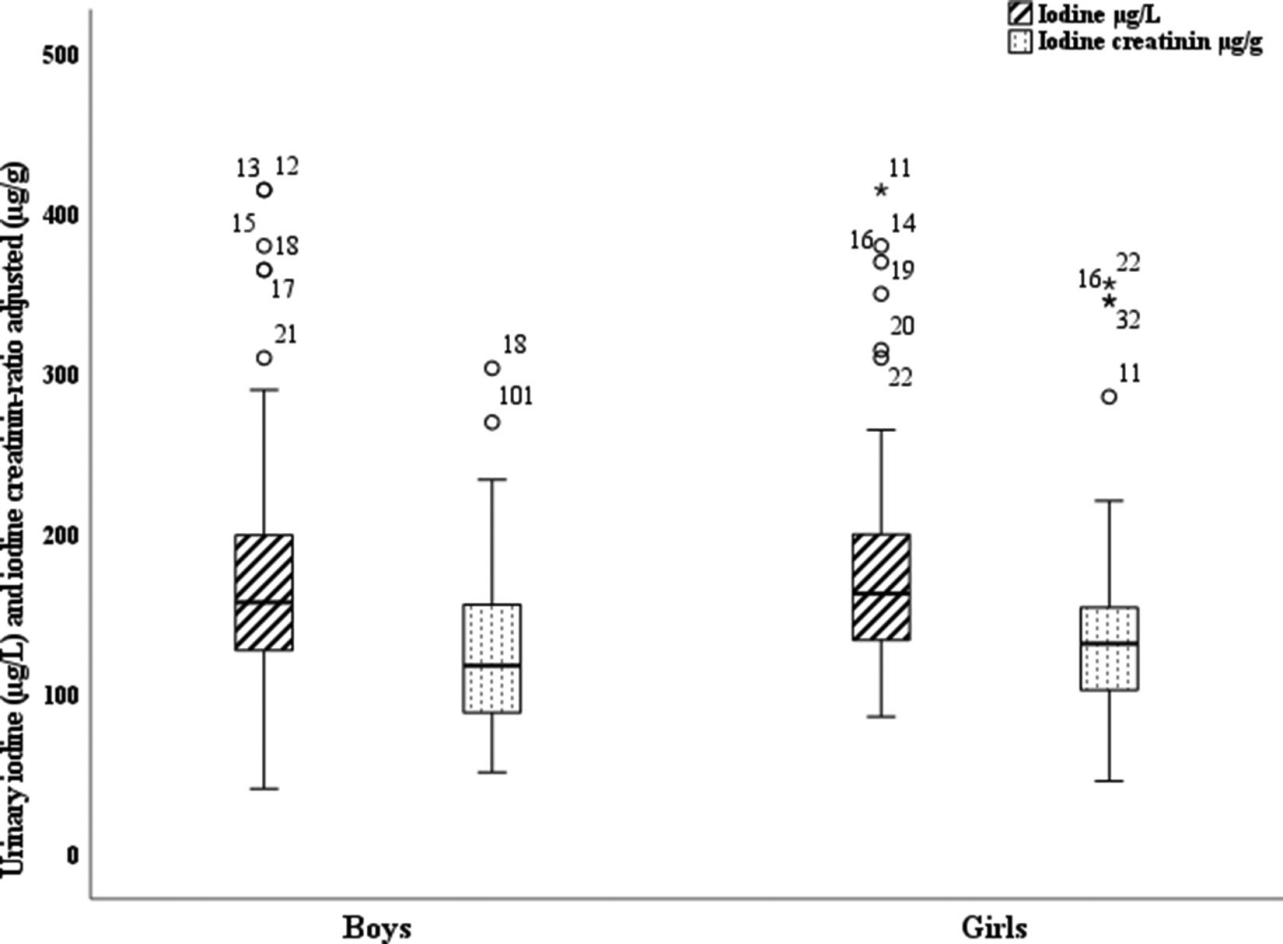
Boxplot illustrating urinary iodine concentrations (μg/l) and the creatinine-adjusted urinary iodine concentration (μg/g) among a population-based sample of 119 Faroese adolescents aged 14 years, omitting ten outliers.

Fig. 1 shows the distribution of UIC among all participants. The median UIC was 166 μg/l (IQR 130–240; bootstrapped 95 % CI 156–184 μg/l) (Table 2). The median UI/Cr was 132 μg/g (IQR 100–172; bootstrapped 95 % CI 120–138 μg/g). Fig. 2 shows a boxplot of UIC and UI/Cr (*n* 119, omitting ten outliers) for each sex. UI/Cr was 134 μg/g in morning samples and 123 μg/g in afternoon samples (*P*_(MW)_ = 0⋅06) (*n* 129). Almost 90 % of the participants living in the capital were examined in the morning compared with 26 % of rural participants, *P*_(Chi)_ < 0⋅001.

**Table 2.** The urinary iodine concentration (μg/l) and the urinary iodine excretion after adjustment for dilution by creatinine excretion (μg/g) and according to sex, age, demographic, lifestyle characteristics and fish frequency meals (*n* 129)

			UIC (μg/l)				UI/Cr (μg/g)			
Variables	Groups	*n*^†^	Median	25–75 Percentiles	95 % CI^‡^,^§^	*P*-value^||^	Median	25–75 Percentiles	95 % CI^‡,§^	*P*-value^||^
Overall		129	166	130–240	156–184		132	100–172	120–138	
Sex	Boys	64	160	128–239	150–184	0⋅6	124	91–178	110–137	0⋅2
	Girls	65	169	141–243	155–190		134	104–167	123–149	
Residence	Capital	48	173	124–265	152–193	0⋅6	132	101–195	114–151	0⋅6
	Rural	80	160	132–216	153–185		129	91–159	118–144	
BMI*	Healthy weight (<85th p)	110	166	130–231	156–184	0⋅8	130	94–164	118–139	0⋅2
	Overweight and obesity (≥85th p)	19	173	123–310	138–260		134	105–340	111–225	
Smoking	Daily and occasionally	8	222	119–335	116–350	0⋅6	142	116–257	110–270	0⋅3
	Never	119	163	130–230	155–184		131	99–165	120–137	
Fish dinners	Up to 2 per week	57	159	125–191	152–173	0⋅08	120	79–145	102–132	0⋅006
	Three or more	72	184	138–286	153–200		140	106–189	128–154	

* BMI, body mass index, kilograms divided by height in meters, squared.

† *n* sample size.

‡ CI, confidence interval.

§ 95 % CI based on 1000 Bootstrap samples.

|| *P*-value assessed by using non-parametric Mann–Whitney *U* test.

### The determinants of iodine nutrition

There was weak evidence to support an association between high fish intake (≥3 meals per week) and UIC (*P*_(MW)_ = 0⋅08), and no association between UIC and the number of meat dinners, whale dinners or whale blubber consumption (data not shown in the table). The remaining variables presented in Table 2 did not correlate with UIC or UI/Cr.

Only fish intake 3+ times per week showed a significant relationship with UIC, and UIC was 24 % lower per unit change in fish intake (fractional change 1⋅24) in the univariate analysis. Multivariate analysis with backward elimination showed that weekly fish intake 3+ was associated with 26 % lower UIC (fractional change 1⋅26; *P* = 0⋅03; *R*^2^ = 0⋅04). The model explained 8 % of the overall variation (*R*^2^ = 0⋅08; *F* = 1⋅8, *P* = 0⋅1).

## Discussion

Early recommendations on iodine nutrition were based on surveys of children, adolescents and pregnant women because these individuals were considered the most vulnerable to iodine deficiency. Recently, adults aged 40+ years living on isles in the North Atlantic were found to have a UIC low within the recommended range^(^^15)^. The adults had a more frequent intake of marine food items than younger individuals^(^^30)^. Hence, we speculate whether the younger generation have a lower iodine intake, and we found it pertinent to evaluate the iodine status in Faroese adolescents. Our study provides the first data on iodine nutrition in a younger population of the Faroe Islands. We found a median UIC of 166 μg/l among 14-years old Faroese and 132 μg/g when adjusted for variation in fluid intake. Additionally, a diurnal variation was detected, suggesting higher UI/Cr in the morning than in the afternoon.

Assessment of iodine status with UIC in spot urine samples is a recommended marker of population iodine status^(^^4,14,31)^. Urinary iodine excretion portrays dietary iodine intake because more than 90 % of dietary iodine is excreted in the urine, and the vast majority within 24 h^(^^32)^. However, urinary iodine is highly variable, and the assessment of individual iodine status from a spot urine sample is unreliable^(^^29)^. The WHO recommends using median UIC when evaluating the iodine status of a population, and a median of 120 samples portrays the iodine nutrition level within ±10 %^(^^29)^. According to the WHO, median UIC below 100 μg/l in a population suggests iodine deficiency, and UIC above 300 μg/l classifies the population as having excessive iodine intake^(^^33)^. The Faroese adolescents in the present study were classified as iodine-replete, according to the WHO. We added an adjustment for variation in fluid intake by the iodine/creatinine ratio, which reduces the number of samples needed by 20 % and increases the reliability of the estimated iodine nutrition^(^^29)^. This adjustment reduced the iodine level slightly, but participants remained iodine-replete.

The Faroe Islands comprise a small, remote island community in the North Atlantic. However, the Faroese dietary peculiarities, such as seabirds, dry lamb and fish, are like those of the Shetland^(^^34)^ and Orkney Islands^(^^35^^)^. Hence, our results may extend to other populations of North Atlantic coastal societies, including Greenland, Iceland, Ireland, Scotland and Norway.

### Food sources and iodised salt

Fish and dairy foods are important sources of population iodine intake^(^^14,36,37)^, and iodine-rich marine food items such as seal, whale meat and blubber have been critical sources of iodine in Arctic people^(^^14^^)^. In line with these reports, the frequency of fish meals appears to be associated with the UIC in our study. Thus, the ongoing nutritional changes with decreasing intake of traditional marine food items emphasise the need for iodine nutrition surveillance. Interestingly, two in five teenagers still had an intake of blubber from marine mammals during urine collection, probably supporting iodine nutrition. However, the urine samples were collected before revising the dietary recommendation restricting pilot whale meat and blubber as human food. Hence, it is fair to speculate that the intake of these food items has decreased, accelerating the dietary transition towards a more western diet and lowering the intake of iodine-rich food items^(^^30)^. The dietary change observed among the Faroese was that fish intake had decreased from 70 to 40 g/d between 1982^(^^38)^ and 2001^(^^39^^)^. The 1982-survey among adults found a daily fish intake of 78 g, suggesting a mean iodine intake of 244 μg (–24 μg females; +32 μg males (*n* 331))^(^^38^^)^. Recent Faroese data on fish intake illustrated an even lower consumption, especially among younger people^(^^30^^)^, which conforms to our finding of lower iodine intake. These changes emphasise the need for continuous monitoring of iodine nutrition to settle the need for salt iodisation legislation and enforcement.

Much of Europe is iodine-deficient^(^^40–42^^)^. Information about iodine intake level, use of iodine-containing supplements and thyroid disease are required to evaluate the need for an iodisation programme. At least 126 countries have implemented mandatory legislation for salt iodisation, and at least 21 more have some form of voluntary legislation^(^^42^^)^. Universal salt iodisation is the best intervention to provide adequate iodine to the population to eliminate iodine deficiency disorders^(^^2,4^^)^. The programme should be well managed and designed for the national situation. Accordingly, the Danish iodine fortification programme with mandatory iodisation of all salt was implemented in 2000, which led to a rise in median UIC from 61 to 101 μg/l^(^^27^^)^. Most salt used in the Faroe Islands originates from Denmark. Thus, the Danish salt iodisation programme influenced the Faroese. However, non-iodised salt remained available for table salt and for commercial bread production in the Faroe Islands. Thus, the influence of the salt iodisation programme on the Faroese remains uncertain, and our report on UIC in urine samples collected following the implementation of the iodine fortification programme helps to close this knowledge gap.

### Diurnal variation in iodine nutrition

The median creatinine-adjusted urinary iodine was higher in the morning than in the afternoon urine samples. Diurnal variation in iodine excretion has been described previously^(^^43^^)^. It is important to notice such diurnal differences as they may affect estimates of iodine intake levels. If all samplings are morning spot urine samples, it may cause an underestimation of iodine excretion, with the discrepancy increasing with rising iodine excretion levels^(^^12^^)^. The present study subsequently noted that time-of-day for participating and collecting urine samples differed with the residence. Such a difference could influence the interpretation of results, and we thus refrain from further evaluation of differences between capital and rural participants.

### Strengths and limitations

The data on iodine nutrition among people living on remote islands in the North Atlantic Ocean is unique. The samples were collected and analysed 20 years ago. Still, they are of interest because samples were collected with the mandatory salt iodisation initiated in Denmark year 2000. This salt iodisation is believed to influence the iodine nutrition among the Faroese as most edible salt is imported from Denmark. With data from this study, we aimed to establish a baseline for monitoring iodine and dietary habits in the Faroe Islands. A survey of iodine nutrition in pregnant women has been commenced to extend the findings of the present study.

Moreover, there are limited data on female adolescents who are more vulnerable to the consequences of iodine deficiency in their near-future pregnancies^(^^44^^)^. The nationwide population-based design supports that the data are representative of this age group, and the 65 girls included had a UIC of 169 μg/l, suggesting that the actual UIC was between 127 and 211 μg/l^(^^29)^. Finally, the element iodine is stable with storage^(^^45^^)^, and our evaluation of urinary iodine collection and measurement are in keeping with procedures recommended for quality control^(^^25^^)^.

The study population was limited. However, the Faroese population is small, and the number of participants was sufficient to portray the iodine nutrition level. Thus, the number of participants led to a 90 % precision of the estimated iodine nutrition level with 95 % confidence^(^^29^^)^, and our finding of a median UIC of 166 μg/l confirms a true UIC in the interval of 156–184 μg/l.

The urine samples were collected and initially donated for biobank storage, and the present study is a secondary analysis of these data, and publication is warranted. The questionnaire focused on pollutants in Faroese foods and did not include iodine-specific questions such as the use of iodised salt in the household. Nevertheless, the mandatory salt iodisation in Denmark may have affected the Faroe Islands, and monitoring should be implemented.

## Conclusion

Our nationwide study demonstrated that Faroese teenagers were iodine-replete at the turn of the millennium. The sampling was conducted around the time of implementing iodine fortification of salt in Denmark, which included the Faroese voluntarily. Still, our data on the impact of reduced fish intake on UIC emphasise the need for continuous monitoring of iodine nutrition. Moreover, it may be speculated that the Faroese have adapted to a formerly high iodine intake from iodine-rich foods and surveying the occurrence of iodine deficiency disorders is warranted. If follow-up on the present findings confirms a decline in iodine nutrition, mandatory salt iodisation may be needed to ensure continued adequate iodine intake among Faroese. These points highlight the need for continuous monitoring of iodine nutrition.
